# A Multi-Analytical Approach to Investigate the Polychrome Clay Sculpture in Qinglian Temple of Jincheng, China

**DOI:** 10.3390/ma15165470

**Published:** 2022-08-09

**Authors:** Jingyi Shen, Li Li, Dandan Zhang, Shaohua Dong, Jiankai Xiang, Nuo Xu

**Affiliations:** 1 School of History and Culture, Shandong University, Jinan 250100, China; 2 Shaanxi Institute for the Preservation of Cultural Heritage, Xi’an 710075, China; 3 Xi’an Museum, Xi’an 710075, China

**Keywords:** polychrome clay sculpture, Qinglian Temple, materials science and techniques, Micro-Raman, SEM-EDS, XRD

## Abstract

This article presents an integrated analytical method to investigate the polychrome clay sculptures of the Qinglian Temple in Jincheng City, Shanxi Province, China. Digital microscopy, SEM-EDS, XRD, Herzberg stain, Micro-Raman spectroscopy and FT-IR were selected to identify the raw materials and techniques used to produce the ground clay layer, the white powder layer and the mineral pigment of the polychrome clay sculptures. The investigation shows that the clay used to make the coarse and fine clay layer is consistent. However, different kinds of fibres were found mixed in the coarse and fine clay layers: wheat straw was the main fibre used in coarse clay layer, while the bast fibres, including ramie, kenaf and sisal hemp, were used as the fibre supports in the fine clay layers due to their useful properties. The white powder layer was made of a mixture of kaolinite and gypsum. For the mineral pigments, it principally contained red (hematite, minium or a mixture of minium and cinnabar), green (atacamite and atacamite), blue (azurite), yellow (yellow ochre), black (amorphous carbon) and white (the mixture of kaolinite and gypsum). Additionally, a gilding technique and multiple paint layers also typified many pigment areas. This work has furthered understanding of the materials and techniques used in making the sculptures of the Qinglian Temple and has clear implications for the restoration and conservation treatments on these kinds of ancient painted clay sculptures.

## 1. Introduction

**Figure 1:**
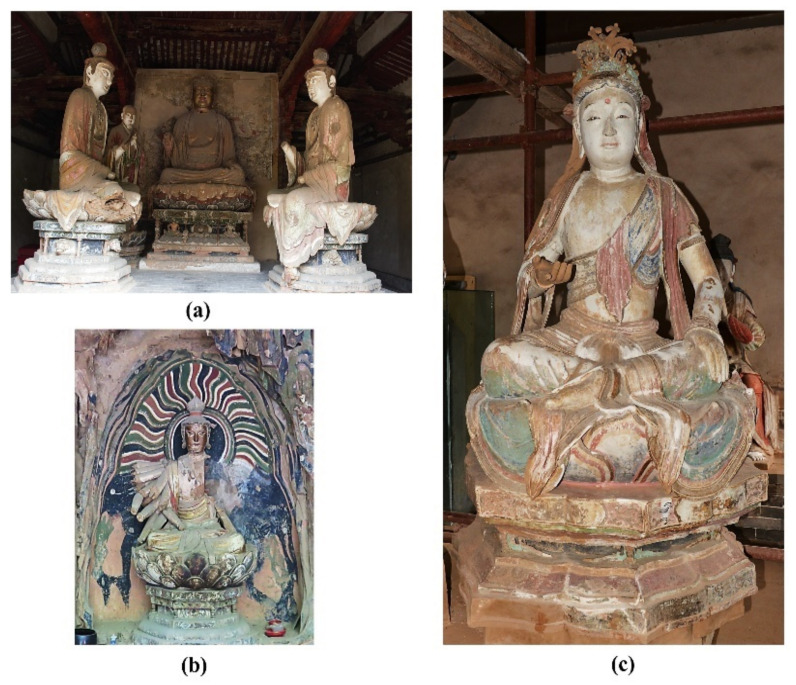
The halls and sculptures in the Qinglian Temple: (**a**) the interior of the Shangsibei Hall; (**b**) Sculpture of a Thousand Hand Guanyin statue in the Shangsibei Hall; (**c**) Sculpture of Bodhisattva Manjusri in the Xiasinan Hall.

Qinglian Temple, one of the earliest temples created in China by the Sukhavati Sect, is located in the southeast of Jincheng City, Shanxi Province. Qinglian Temple is listed as a Key Cultural Relics Site under the State Protection act due to its unique Chinese architecture and, perhaps more importantly, the 66 exquisite polychrome clay sculptures distributed throughout its six halls. At present, throughout China, only 70 painted clay sculptures produced in the Tang Dynasty (CE 618–907) are known to have been preserved; these are distributed across three temples, and six of these are in the Xiasibei Hall (also known as Mile Hall) of Qinglian Temple. In addition, 51 of the sculptures of Qinglian Temple were produced in the Northern Song Dynasty (CE 960–1127), and these are housed across the Xiasinan Hall (Shijia Hall), the Luohan Hall, the Dizang Hall and the Shangsibei Hall (also named as Shijia Hall). A further seven sculptures made in the Ming Dynasty are also located in the Sanfo Hall. However, while the original structures of all these painted clay sculptures at Qinglian Temple have been preserved, most of the pigment layers were renewed in the early period of the Qing Dynasty (CE 1636–1912). Several halls and sculptures in the Qinglian Temple are shown in Figure 1.

The sculptures at Qinglian Temple are mainly Buddhist statues, featuring images of the Buddha, Bodhisattva, Arhat, and the god generals. Relevant sources [1,2] show all these clay-based sculptures as essentially being made using the similar steps: a wood skeleton for each sculpture was made based on its general shape; this skeleton was then wrapped with reed poles fixed with hemp ropes, then an initial clay layer made of a mixture of clay, sand and fibre was applied as a base. The surface was then whitened and painted, with complex mineral pigments used to obtain the required exquisite patterns.

The painted clay sculptures of Qinglian Temple have been identified as valuable contributions to national cultural heritage due to their artistic appearance, exquisite traditional craftsmanship and significant historical value. However, various natural and human factors have led to a large number of these sculptures experiencing significant damage, mainly with regard to the flaking and shedding of the pigment layer and the cracking and salt efflorescence of the ground clay layer. In such situations, urgent preservation and restoration is required, making it important to reveal the details of the ancient materials and techniques used to produce these polychrome clay sculptures using scientific and analytical methods; such knowledge is essential in order to develop effective protection measures.

Certain analytical methods have already been proven to be effective in the scientific analysis of the various structures of ancient polychromic artworks, including surface or cross-section observation using optical microscopy [3], digital microscopy [4] and scanning electron microscopy (SEM) [5]; X-ray diffraction (XRD) [6] and SEM [7] are particularly beneficial for accurately identifying the crystalline structures and microstructure of the clay ground layer while a combination of Herzberg stain and optical microscopy is commonly used to identify the fibre sources in ground clay layers [8]; particle induced X-ray emission spectroscopy (PIXE) [9], X-ray fluorescence (XRF) [10] or energy dispersive spectroscopy (EDS) [11] have also been used to analyse the elements of the relevant mineral pigments, and a combination of Raman spectroscopy (RS) [12,13], Fourier transform infrared spectroscopy (FT-IR) [12,13] and XRD [7] have also been found to be beneficial for accurately identifying pigments.

The aim of this paper is to investigate the materials and relevant manufacturing techniques used to make the different structures of the polychrome clay sculptures in Qinglian Temple by applying various analytical techniques, including digital microscope, SEM-EDS, XRD, Herzberg stain, Micro-Raman and FT-IR. The research results are useful not only to achieve a comprehensive understanding of the original materials and the history, culture and artistic value behind the painted clay sculptures of Qinglian Temple, but also to provide scientific information for their future conservation and restoration.

## 2. Materials and Methods

### 2.1. Sample Information

This study selected the samples from the damaged areas of the sculptures or those which had been fallen off the sculptures. The samples mainly include soil and fibre in ground clay layers, samples of white powder layers and pigment samples of sculptures located in different halls. The sample details are mainly given in Table 1 and Table 2.

### 2.2. Analytical Methods

#### 2.2.1. Digital Microscopy (DM) Analysis

Digital microscopy (KH-7700, HIROX, Japan, the instrument is located in Xi’an, China) was applied in situ observation of polychrome clay sculptures to examine their layer structure, and also used to observe the surfaces and cross-sections of polychrome clay samples in macroscopic morphology. The polychrome samples were magnified from 20× to 200×.

#### 2.2.2. X-ray Diffractometer (XRD) Analysis

The clay samples from clay ground layer were tested by XRD (RIGAKU SmartLab, the instrument is located in Xi’an, China) for their phase analysis. A 2θ range of 10 to 70 degrees was used, with the detector type being a D/teX Ultra 250 silicon strip detector device. A tube voltage of 30 kV with a current of 300 mA was applied.

#### 2.2.3. Scanning Electron Microscopy (SEM) with Energy Dispersive Spectroscopy (EDS) Analysis

A model EVO-250 SEM (Zeiss), coupled with an Oxford Instruments X-MAX20 EDS system (the instrument is located in Xi’an, China), was selected to examine the microstructure of clay ground samples and the elements of pigment samples. The acceleration voltage used was 20 keV, and working distance was 8 mm. SEM backscattered electron (BSE) images of the clay ground layer were recorded for their microstructure observation. For the element analysis of pigment samples. The size analysed by EDS is around 200 μm × 300 μm, with test time of 90 s. Oxford Instruments standards were applied to quantify elements.

#### 2.2.4. Micro-Raman Spectroscopy Analysis

The pigment and white powder samples were analysed via Micro-Raman spectroscopy (Scientific XploRA PLUS, HORIBA, the instrument is located in Beijing, China) with a 1200 grooves/mm grating and a CCD detector. Point measurements were performed using an argon gas laser at 532/785 nm and Olympus 50× objectives were applied. The scanning time varied between 10 and 60 s depending on each sample separately.

#### 2.2.5. FT-IR Spectroscopy Analysis

FT-IR, as a complementary analytical techniques approach for Micro-Raman spectroscopy analyses, was applied only in two white powder layer samples and one white pigment sample to determine their raw material sources, with the reason that the Micro-Raman results of these three samples were limited. FT-IR was collected by a Fourier transform infrared spectrometer (iN10 MX, NICOLET, USA, the instrument is located in Xi’an, China) using the KBr tablet method. The spectra were set at 4000–400 cm^−^^1^ with a resolution of 4 cm^−^^1^ using 128 scans.

#### 2.2.6. Herzberg Stain

Herzberg stain was applied to identify the fibre type used in the clay ground layer of polychrome clay sculptures. Different fibres are dyed into various colours due to the difference of the quantity of lignin within the fibre [14]. Lignin-free fibres (such as hemp, ramie and cotton) are dyed in red-wine colour. With the increase of lignin ratio, the colour of fibres changes from red-wine to grey- bluish to grey-yellow-greenish and finally to totally yellow (such as bamboo with high lignin content) [8]. The Herzberg reagent used in this study was prepared by TAPPI standard, which is mixing zinc chloride solution (dissolve 50 g ZnCl_2_ in 25 ml distilled water) and iodine solution (dissolve 0.25 g I_2_ and 5.25 g KI in 12.5 ml distilled water) [8,14]. Fibre samples, which were selected from the clay ground layer, were stained with Hertzberg reagent, then were identified and photographed using a ZEISS Scope A.1 polarised light microscope.

## 3. Results and Discussion

**Figure 2:**
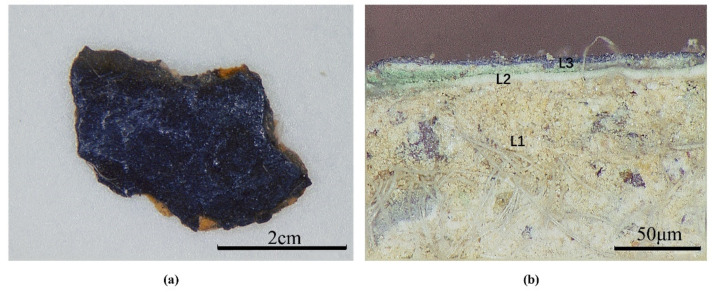
Digital microscopy (DM) of sample SJD-24 from the sculpture of Sakyamuni Buddha in the Xiasinan Hall: (**a**) surface; (**b**) cross-section (L1, clay ground layer; L2, white powder layer; L3, pigment layer).

Digital microscopy images (Figure 2a,b) of the sample SJD-24 and its cross-section show that the polychrome clay sculptures at Qinglian Temple can be classified into three main layers: a clay layer, a white powder layer and a pigment layer. This confirms that the sculptures at Qinglian Temple were made using traditional Chinese sculpting technology, which involves the application of a clay layer on a wood skeleton for shaping, with white powder then used to level and whiten the surface before mineral pigments and gilding are added to develop the required exquisite patterns.

**Figure 3:**
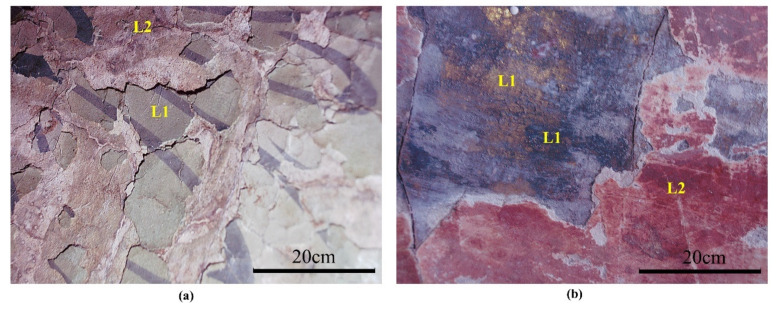
The in situ microphotographs of multiple paint layers: (**a**) at the sculpture of Ananda Buddha in the Xiasibei Hall (XSBD); (**b**) at the sculpture of Bodhisattva Manjusri in the Xiasinan Hall (XSND).

In addition, the in situ digital microphotographs (Figure 3) revealed multiple paint layers of the sculptures. At least two paint layers were found for each of the sculptures, which, from inside to outside, were green and black pigment (L1)–white powder layer–red pigment (L2) for the Ananda Buddha in Figure 3a and gilding and black pigment (L1)–white powder layer–red pigment (L2) for the Bodhisattva Manjusri in Figure 3b. Each L2 pigment layer was applied after the white powder layer was added on the original paint layer (L1), which implies that these multiple paint layers of the sculptures are the result of historical repainting. Repainting of seriously damaged sculptures was common in ancient China, and the craftsmen usually applied a white powder layer or a layer of paper to cover the original paint layer before repainting [2].

The raw materials and relevant techniques determined within the main layered structures of the sculptures are discussed in more detail below.

### 3.1. Clay Ground Layer

**Figure 4:**
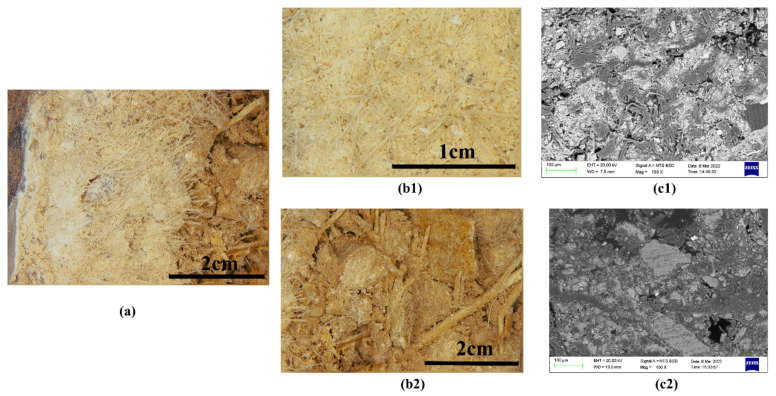
Coarse and fine clay layers of sample XSBD-7 from the sculpture of Sakyamuni Buddha in the Xiasibei Hall: (**a**) digital microscopy (DM) of cross-section; (**b1**) fine clay layer; (**b2**) coarse clay layer; (c) SEM backscattered electron mode images of fine (**c1**) and coarse clay layer (**c2**).

Digital microscopy images (Figure 4a) of the clay ground sample XSBD-7 indicates that it can be further divided into two parts, being a coarse and fine clay layer, respectively. Figure 4b,c shows that the fine clay layer is both finer and denser, being mixed with more slender fibres; however, these fibre types are difficult to identify. The coarse clay layer underlying the fine clay layer is distributed in flakes and blocks, with larger grains of sand and lower concentration of binding, as well as being obviously mixed with identifiable wheat straw fibres.

#### 3.1.1. Clay

The clay ground layers of the polychrome clay sculptures from Xiasibei Hall (XSBD), Xiasinan Hall (XSND) and Sanfo Hall (SFD) were produced in the Tang, Song and Ming dynasties respectively. Five coarse clay samples and seven fine clay samples from sculptures of these three halls were thus selected to identify the raw materials used.

**Figure 5:**
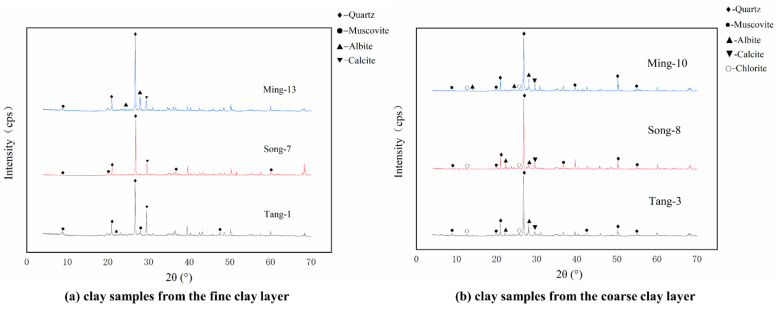
Mineral composition of coarse and fine clay by XRD: Tang-1 and 3 were collected from sculptures in XSBD (Tang dynasty); Song-7 and 8 were collected from sculptures in XSND (Song dynasty); Ming-10 and 13 were collected from sculptures in SFD (Ming dynasty).

The XRD analytical results of all samples are listed in Table 1, and some representative XRD patterns are shown in Figure 5. The results suggest that quartz (SiO_2_), albite (Na(AlSi_3_O_8_)) and calcite (CaCO_3_) are the primary crystalline phases in both the coarse and fine clay layers; moreover, some muscovite ((KAl_2_(AlSi_3_O_10_) (OH)_2_) is also found in both the coarse and fine clay layers. The crystalline phases of the clay used to make the base clay layers of the sculptures across these different ancient Chinese dynasties also appear to be similar, suggesting that the craftsmen of the Tang, Song and Ming dynasties may have focused on the use of local clay to make these painted clay sculptures. According to the literature, the coarse and fine clay layers of sculptures were both generally made by mixing clay, sand and fibre together [2], and in this study, the clay used to make the coarse and fine clay layers appears to be consistent. The reasons for the different microstructures of the coarse and fine layers are thus mainly found in the different proportions of clay and sand, the different particle sizes of clay and sand selected for each layer and the different fibres added.

#### 3.1.2. Fibres

**Figure 6:**
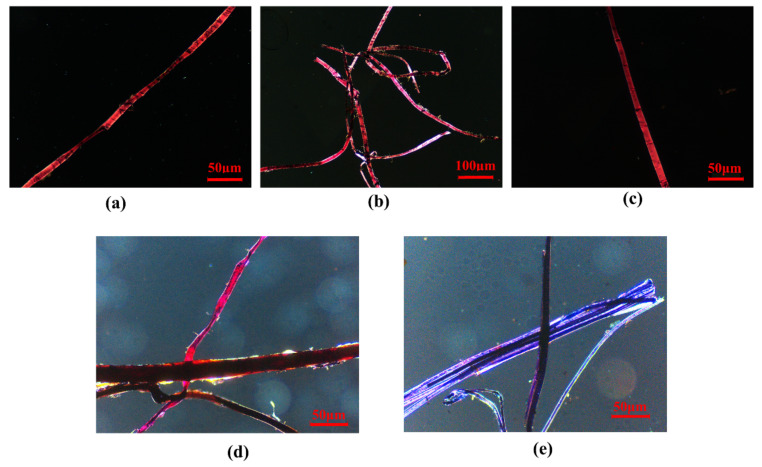
Morphology of paper fibres: (**a**) XSBD-F1; (**b**) XSBD-F2; (**c**) XSND-F4; (**d**) XSND-F5; (**e**) XSBD-F3.

As shown in Figure 4(b2), wheat straw is the main fibre type used in coarse clay layer. Other fibres, whose sources are more difficult to determine, were also observed, mainly within in the fine clay layer. Four fibre samples from the fine clay layer and one fibre sample from coarse clay layer were thus selected and identified using the Herzberg stain method. The morphology figures for the stained fibre samples are shown in Figure 6, and the morphological characteristics of the fibres and the related results are listed in Table 2.

Based on fibre identification, the main fibre added to the coarse clay layer was wheat straw, with additional bark in some cases: more specifically, the bark of green sandalwood was identified in this study. For the fine clay layer, bast fibres including ramie, kenaf and sisal hemp were used as fibre sources, though cotton was also identified in the fine clay layer. At present, there is little scientific research on the fibres used in painted clay sculptures, though those identified in this study, including wheat straw, the various bast fibres (ramie, kenaf and sisal hemp) and cotton are commonly used in Oriental papermaking [14]. This study thus suggests that these different kinds of fibres were consciously selected for their varying properties by ancient craftsmen and added to the coarse clay layer and fine clay layer as appropriate.

Wheat straw fibre has the physical properties of low density, a multi-fibre structure, freeze–thaw resistance and high vertical plane tensile strength, and these characteristics mean that the addition of wheat straw fibre could enhance the bonding strength of the clay layer, reducing the overall weight of the final clay sculpture as well as reducing shrinkage and cracking of the clay layer. Cotton and bast fibres are slender and long, and, when mixed with clay, they may be better integrated than wheat straw fibre, making the clay more delicate as well as having minimal impact on the appearance of the final clay sculpture. Cotton and bast fibres are thus more suitable for fine clay layers.

### 3.2. White Powder Layer

**Figure 7:**
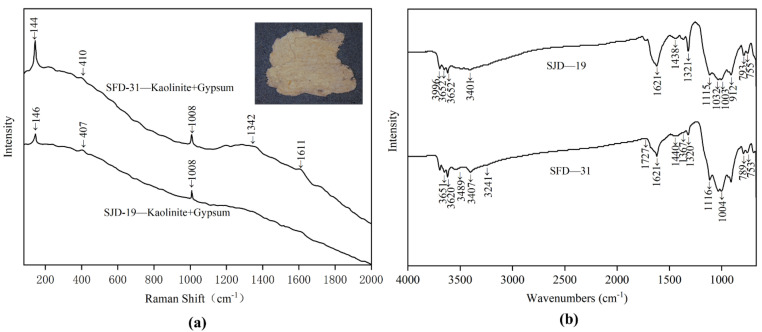
Micro-Raman spectroscopy (**a**) and FT-IR spectra (**b**) of white powder samples SFD-31 and SJD-19.

The white powder layer is sandwiched between the clay ground layer and the pigment layer, levelling the clay ground layer and covering its inherent colour to facilitate the application of pigments. Two samples (SJD-19, SFD-31) of white powder layer were analysed to determine their raw materials, and their Raman spectra, shown in Figure 7b, indicated that they were formed of a mixture of kaolinite and gypsum. The weak typical Raman peaks of 144/146, 407/410 cm^−^^1^ indicated the presence of kaolinite [15] while the peaks of 1008 cm^−^^1^ were attributed to gypsum, based on the bending vibration of SO_4_^2−^. The Raman peaks of 1372 and 1611 cm^−^^1^ are characteristic of the D-band and G-band of amorphous carbon [16], which may indicate the presence of contamination or impurities. Possibly due to the strong fluorescence arising from other agents or the aging of the white powder layer, the typical Raman peaks of these two samples were somewhat unclear, however. The samples were thus also analysed using FT-IR spectra to confirm the Raman results. As shown in Figure 7b, the peaks of the FT-IR spectra at 3544, 3488, 3407, 3401, 3241, 1621, 1116, and 1115 cm^−^^1^ confirm the present of gypsum. Besides, the peaks at 3695, 3696, 3652, 3651, and 3620 cm^−1^ were assigned to O-H stretching, while those at 1033, 1032, 1004, and 1003 cm^−^^1^ were assumed to belong to Si-O stretching. The peaks at 936 and 912 cm^−^^1^ were then attributed to Al-OH stretching and Al-OH bending, respectively, with those at 793, 789, 755, 753, and 696 cm^−1^ assigned to Si-O, Si-Si and Si-Al stretching. All these peaks were identified as belonging to the the 1: 1 type of clay minerals.

Raman results show that the main component of the white powder layer is kaolinite. Kaolinite is the most prominent member of the 1: 1 type of clay minerals identified by FT-IR. Kaolin, with kaolinite as the main component, is a commonly used base whitening material in ancient times. In this situation, a mixture of kaolin and gypsum was thus identified as the main raw material used to make the white powder layer of these clay sculptures in Qinglian Temple. However, previously published research, chalk [7], calcite [17] and lead white [17] seems to be a more commonly used material for the white powder layer of ancient painted clay sculptures and wall paintings.

### 3.3. Pigments

The major colours of the sculptures are gold, red, blue, green, yellow, white, and black. Table 3 shows an overview of the pigments identified using SEM–EDS and Raman spectroscopy.

#### 3.3.1. Gold

**Figure 8:**
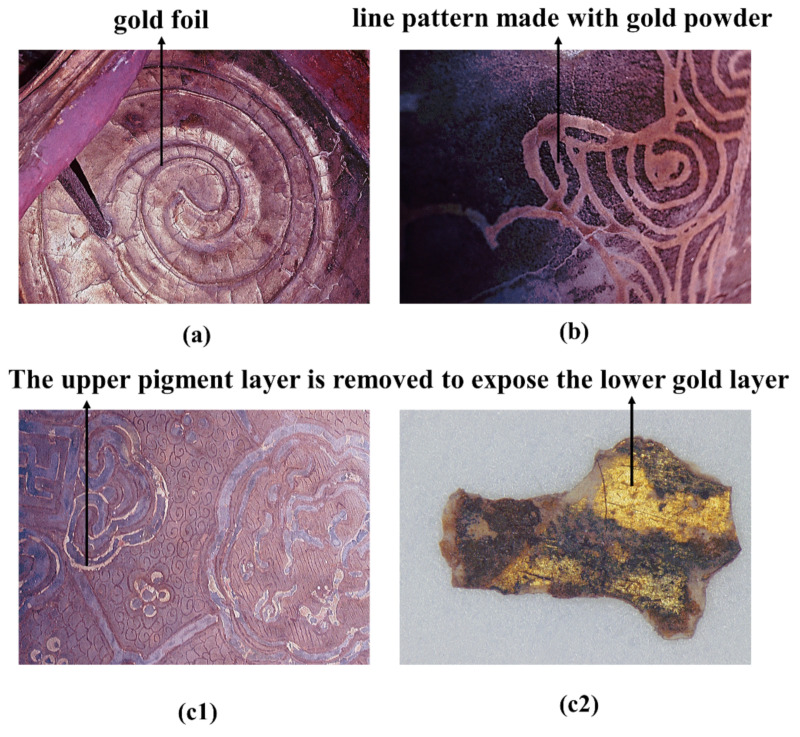
Digital microscopy (DM) of “gilding technique”: (**a**,**b**,**c1**) in situ microphotographs; (**c2**) flaked sample (SFD-33).

Gilding techniques have been used in various cultural relics, and they have been commonly used in Chinese painted clay sculptures since the Song dynasty (CE 960–1279) [1]. The EDS results for the two gold-coloured samples (SFD-33 and SJD-26) confirmed that they had a high gold (Au) content, while also containing other metal elements such as Ag, Pb, Cu and Fe, consistent with the obvious metallic lustre observed under digital microscopy (shown in Figure 8). The results of this study are thus in agreement with the literature, as most relevant studies have demonstrated that, in the gilding process, gold foil or gold powder was mostly used as a surface material, with relatively cheaper metals such as silver, iron, copper and lead powder used as the base material to bolster the shine and colour of the gold surfaces. This method was used to achieve the required effect at a lower cost to manufacture.

The in situ microphotographs, as shown in Figure 8, show three types of gilding techniques. Figure 8a represents the most common type, in which large piece of gold foil was gilded onto a painted surface. This type is mainly decorated on the large area of exposed skin and cassock to create a magnificent appearance. Figure 8b shows the more precise gilding technique, in which gold powder was used to decorate small areas of complex accessories, such as the collars, tassels, belts or decorative patterns on clothes, while Figure 8(c1,c2) represents a special gilding technique that involved applying gold foil or powder as the base and then drawing decorative patterns with pigments above these, with the edge of the paint layer removed according to the desired pattern to reveal the gilding below. This method was used to set off various black, green, red or blue areas with gold lines to great effect. The gold lines exposed using this decoration method are usually very thin, generally less than 3 mm, as shown in Figure 8(c1,c2).

#### 3.3.2. Red

Red is the most common colour of the polychrome clay sculptures, with chromatic variations occurring, from orange-red to very dark shades, that highlight the artistry of the painted patterns. Seven red samples with different hues were thus assessed for this paper.

**Figure 9:**
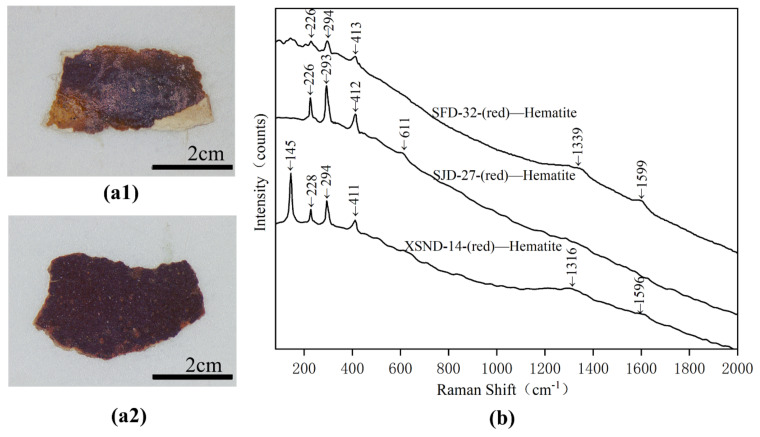
Digital microscopy (DM) of red samples (**a1**) XSND-14; (**a2**) SJD-27) and Micro-Raman spectroscopy (**b**) SFD-32; SJD-27 and XSND-14.

Samples SJD-27, XSND-14 and SFD-32 are of dark red hue, and the EDS results (shown in Table 2) indicate that they have high Fe levels, which is the only element that may present a red colour among the measured elements. Their Raman spectra were shown in Figure 9b, and the Raman bands at 145, 226/228, 293/294 and 411/412/413 cm^−^^1^ had been identified as belonging to hematite (Fe_2_O_3_) [18,19]. The weak Raman peaks at 1316, 1339 and 1596**/**1599 cm^−^^1^, however, were ascribed to carbon black, potentially from the black soot grains left in the pigment samples. The use of red ochre (hematite) as a pigment is an ancient technique, dating back to the palaeolithic era [20]. It was widely used across an array of different kinds of cultural relics, including rock paintings [20], painted pottery [21] and polychrome clay sculptures [17] from different periods.

**Figure 10:**
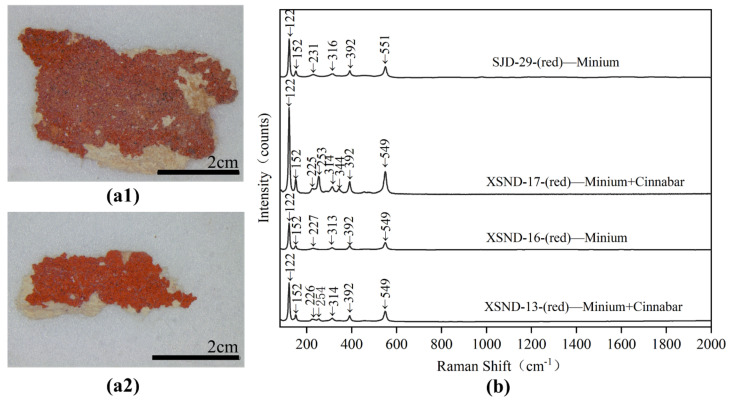
Digital microscopy (DM) of red samples (**a1**) SJD-29; (**a2**) XSND-17 and Micro-Raman spectroscopy (**b**) SJD-29; XSND-17; XSND-16 and XSND-13.

The samples of XSND-13, 16, 17 and SJD-29 were of paler red and even orange-red hues. The EDS results (shown in Table 2) of samples XSND-13 and 17 show that they mainly contain elements such as S, Hg, Pb and O, and their Raman spectra shown in Figure 10b confirm that they are a mixture of minium and cinnabar. Their Raman bands at 122, 152, 225/226, 314, 392 and 549 cm^−^^1^ were ascribed to minium (Pb_3_O_4_) [22], while the absorption at 549 cm^−^^1^ was attributed to the stretching of the Pb-O bond [23,24]. The bands at 253/254 and 344 cm^−^^1^ were identified as belonging to cinnabar (HgS) [22], while the Roman peaks at 253 and 254 cm^−^^1^ were attributed to the stretching vibration of Hg-S bonds [25,26]. For the sample XSND-16 and SJD-29, the main elements being Pb and O, their characteristic Raman bands (Figure 10b) at 122, 152, 227/231, 313/316, 392 and 549**/**551cm^−^^1^, were attributed to minium (Pb_3_O_4_). The Raman results combined with SEM-EDS analysis indicated that the red pigment samples XSND-13, 16, 17 and SJD-29 were thus minimum or a mixture of minium and cinnabar: this is reasonable, as using mixed materials made from two or more pigments in one colour layer to achieve the desired hue is a common traditional technique in Chinese artworks [2,4,27].

#### 3.3.3. Green

Various different green pigments were also detected in the polychrome clay sculpture of Qinglian Temple, including atacamite (Cu_2_(OH)_3_Cl) and malachite (Cu_2_(OH)_2_CO_3_).

**Figure 11:**
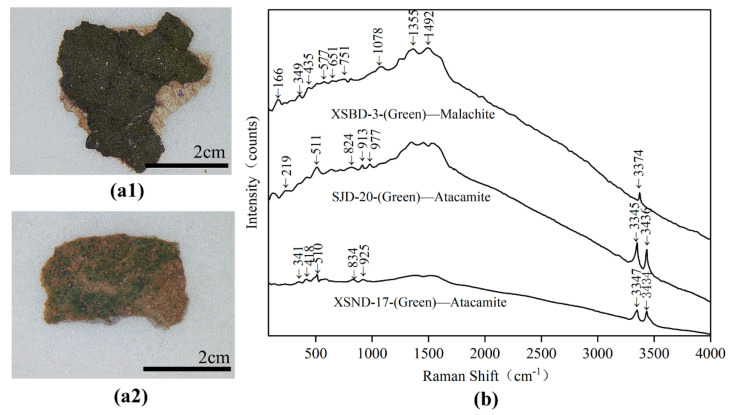
Digital microscopy (DM) of green samples (**a1**) XSND-17; (**a2**) XSBD-3 and Micro-Raman spectroscopy (**b**) XSBD-3; SJD-20 and XSND-17.

The dark green pigments (XSND-17 and SJD-20) were identified as atacamite (Cu_2_(OH)_3_Cl). SEM-EDS analysis (Table 2) showed that they have relatively high levels of Cu and Cl. However, elemental analysis alone cannot rule out the possibility that the Cl may come from environmental impurities rather than the pigment itself. The Raman spectrum, as shown in Figure 11b, supports further investigation. The characteristic Raman bands at 219, 341, 418, 510/511, 824, 834, 913, 925, 977, 3345/3347 and 3434/3436 cm^−^^1^, were ascribed to atacamite (Cu_2_(OH)_3_Cl). In particular, the Raman bands at 824, 834, 913, 925 and 977 cm^−^^1^ were attributed to the bending vibration of O-H and Cu-OH, while the peaks in the range of 219 and 511 cm^−^^1^ were assigned to the vibration modes of O-Cu-O and Cl-Cu-Cl [4,28], and the bands at 3345/3347 and 3434/3436 cm^−^^1^ were assigned to OH stretching [29].

The light green pigment (XSBD-3) was confirmed as malachite (Cu_2_(OH)_2_CO_3_) based on its Raman spectra (seen in Figure 11b) of 166, 349, 435, 1078, 1355, 1492 and 3374 cm^−^^1^, which are the characteristic Raman peaks of malachite [4,30,31]. Its high C, Cu and O content as assessed by SEM-EDS is essentially also in accordance with malachite.

Both malachite and atacamite were widely used mineral pigments in ancient times. In China, malachite’s use as a green pigment can be traced back about 4000 years, to the site of Taosi where it was used to decorate painted pottery [32]. It was also commonly used in polychrome clay sculptures, based on evidence from sites such as the Qianfoya Grottos [33]. Atacamite, another representative pigment used for green colouration, was also widely used in ancient Chinese polychrome clay sculptures and murals after the time of the Five Dynasties (CE709-960) due to the advancements of synthetic technology in that period [4,34]. In ancient China, atacamite was a relatively scarce mineral, with natural green copper ore only been found in the Kangjiltag gold deposits in Xinjiang province [35]. Atacamite was thus usually mixed with other pigments before the time of the Five Dynasties, as seen in the Mogao Grottoes [36] and Yulin Grottoes [34]. In this study, malachite and atacamite were found to decorate different areas of the polychrome clay sculptures, reflecting the conscious selection of different mineral pigments by the ancient craftsmen to present subtle tonal differences to improve artistic expression.

#### 3.3.4. Blue

**Figure 12:**
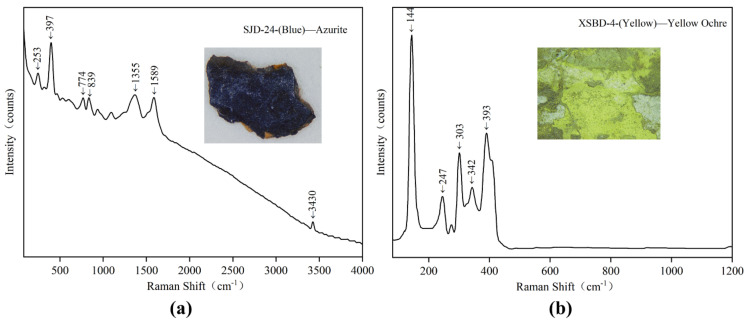
Digital microscopy (DM) and Micro-Raman spectroscopy of samples: (**a**) SJD-24 blue pigment; (**b**) XSBD-4 yellow pigment.

One blue pigment (SJD-24) in the polychrome clay sculpture of Qinglian Temple was detected as azurite (Cu_3_(CO_3_)_2_(OH)_2_. All peaks as seen in Figure 12a between 200 and 1600 cm^−1^ (253, 397, 774, 839, 1355 and 1589 cm^−1^) in the blue sample were characteristic azurite (Cu_3_(CO_3_)_2_(OH)_2_) [35]. The Raman band at 3430cm^−^^1^ was assigned to the OH stretching region [29]. The relatively high Cu, C and O contents within the SEM-EDS confirm the Raman result.

Azurite was used widely as a blue pigment in ancient China, as seen in the Mogao Grottes [36], and at the Longju Temple [35], Huayan Temple [13], and Jingyin Temple [2]. Azurite also was a most important blue mineral pigment in European paintings throughout the Middle Ages and Renaissance [37]. Despite its relatively high price, azurite was so extensively used as a blue pigment in ancient times because of its chemically stable [11,13,37,38]. In this work, the azurite pigments used in Qinglian Temple were also noted to suffer very little from any apparent aging phenomena, remaining highly vivid.

#### 3.3.5. Yellow

The yellow pigment (XSBD-4) was identified as yellow ochre. A Micro-Raman study as seen in Figure 12b of the sample showed peaks at 144, 247, 303, 342 and 393 cm^−1^, all of which were assigned to yellow ochre. The Fe, O, Ca, S, Si, Al and K content, as assessed by SEM-EDS, also supported the presence of yellow ochre. Natural yellow ochre, also named as limonite, is hydrated iron hydroxide (FeO (OH) · nH_2_O), which is composed of a mixture of several iron-bearing minerals, the main component of which is goethite [39]. Goethite widely exists in many rocks, soils and ochre deposits [39], so the mineral with goethite as the main component is the most widely applied yellow pigment in ancient times. Minerals such as feldspar, quartz, dolomite and other carbonate minerals usually also exist in ochre as accompanying minerals [40], which could explain the elements of Ca, S, Si, Al and K identified in sample XSBD-4. Yellow ochre is normally prepared by washing to remove impurities.

#### 3.3.6. Black

**Figure 13:**
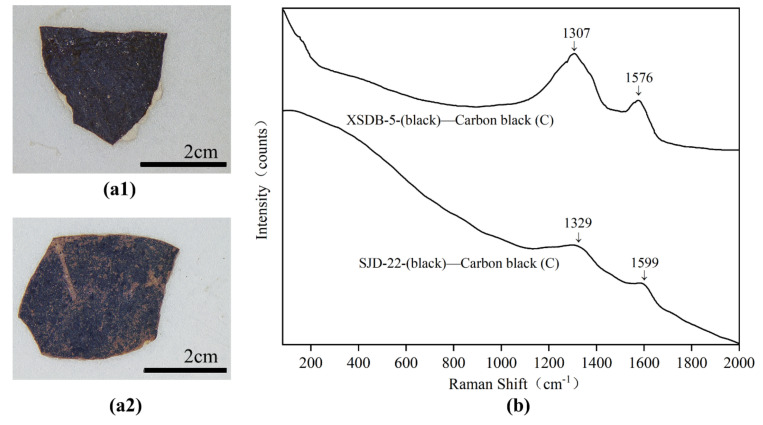
Digital microscopy (DM) of black samples (**a1**) SJD-2; (**a2**) XSBD-5 and Micro-Raman spectroscopy (**b**) SJD-2; XSBD-5.

For black samples (SJD-22 and XSBD-5), the main typical Raman peaks shown in Figure 13 were 1307, 1329, 1576 and 1599 cm^−^^1^_,_ which are the characteristic peaks of D-band and G-band of amorphous carbon [19]. SEM–EDS results confirmed that high levels of C were detected in these two samples. Amorphous carbon black has been widely detected in ancient polychrome relics, including painted sculptures excavated from tombs [16,19], wall paintings [41,42] and on painted sculptures from temples [4,35].

#### 3.3.7. White

**Figure 14:**
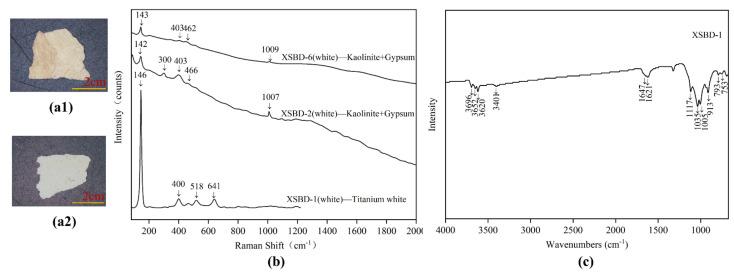
Digital microscopy (DM) of white pigment samples (**a1**) XSBD-1; (**a2**) XSBD-6, Micro-Raman spectroscopy; (**b**) XSBD-1; XSBD-2; XSBD-6 and FT-IR spectra; (**c**) XSBD-1.

Two white pigment samples (XSBD-1 and XSBD-2) were analysed, and these were shown to be a mixture of kaolinite and gypsum, consistent with the raw materials used to produce the white powder layer as discussed above. As shown in Figure 14b, the weak typical Raman peaks of 142/143, 300, 403/404, 462, 466 cm^−^^1^ were associated with kaolinite, while the peaks of 1007/1009 cm^−^^1^ were assigned to gypsum. The FT-IR spectra of sample XSBD-1 also confirmed the Raman results. On the polychrome painted clay sculptures of Buddha, the large areas of the face, arms, legs, and back were always be decorated with white pigment. Based on this study, it appears that perhaps to simplify the process, the raw materials used for the white pigment layer and the white powder layer were the same, effectively making the white pigment a thick layer of white powder layer undecorated with other mineral pigments such as lead white, which was otherwise the most commonly used ancient white pigment until the 19th century [37].

Other whiter and newer pigments were observed in some areas, and a sample (XSBD-6) was obtained to analyse these. As shown in Figure 14b, the main typical Raman peaks in the sample were 146, 400, 518 and 641 cm^−1^, which may indicate the use of titanium white. The relatively high Ti content as assessed using SEM-EDS also confirms this result. Titanium white pigment is mainly extracted from titanium ore and rutile, which were widely used for white colouring from the beginning of the 20th century. This titanium white may thus indicate the materials used in more modern restoration attempts.

## 4. Conclusions

A comprehensive understanding of the raw materials and techniques used to manufacture cultural relics is the premise for their scientific protection and restoration, because only in this way can we select the suitable traditional restoration materials, and then formulate the optimal protection scheme. In this respect, this research has not only furthered understanding of the materials and crafts used in making the sculptures seen at Qinglian Temple based on the application of a comprehensive scientific analysis, but also has provided a scientific foundation for the development of effective conservation schemes.

The results confirmed that the painted sculptures of Qinglian Temple are built up of a coarse clay layer, a fine clay layer, and a white powder layer, finished with a pigment layer. For the ground clay layer, the previous scientific research on painted clay sculptures were few comparative studies on their coarse and fine clay layers, especially the types of fibres doped in them. With respect to Qinglian Temple painted clay sculpture in this study, we found that, compared to the coarse clay layer, the fine clay layer is both finer and denser, though the clay type used to make both layers is consistent. However, different kinds of fibres were consciously selected for their varying properties by ancient craftsmen and added to the coarse clay layer and fine clay layer as appropriate: wheat straw was the main fibre used in coarse clay layer, while the bast fibres, including ramie, kenaf and sisal hemp, were used as the fibre supports in the fine clay layers due to their useful properties.

For the white powder layers, the sculptures of Qinglian Temple were found to made of a mixture of kaolinite and gypsum. Compared with chalk, limestone and lead white, a mixture of kaolinite and gypsum is not a commonly used raw material in the painted clay sculptures production. This presents the uniqueness of the materials selected in the production of Qinglian Temple polychrome clay sculptures.

For the pigment layers, the mineral pigment decoration principally contained hematite, miniu, or a mixture of minium and cinnabar in the red sections; atacamite and atacamite in the green sections, azurite for the blue, amorphous carbon for the black, a mixture of kaolinite and gypsum for white and yellow ochre. Additionally, a gilding technique and multiple paint layers also typified many pigment areas.

## Figures and Tables

**Table 1:** Mineral composition of coarse and fine clay by XRD.

Sample No.	Position	Sculpture	Mineral Composition by XRD
Tang-1	fine clay layer	Bodhisattva Samantabhadra in XSBD (Tang dynasty)	quartz, muscovite, albite, calcite
Tang-2		Bodhisattva Manjusri in XSBD (Tang dynasty)	quartz, muscovite, albite, calcite, chlorite
Song-7		Sakyamuni Buddha in XSND (Song dynasty)	quartz, muscovite, albite, calcite
Song-9		Bodhisattva Manjusri in XSND (Song dynasty)	quartz, muscovite, albite, calcite
Song-11		Bodhisattva Manjusri in XSND (Song dynasty)	quartz, muscovite, albite, calcite
Ming-12		Sakyamuni Buddha in SFD (Ming dynasty)	quartz, muscovite, albite, calcite, chlorite
Ming-13		Sakyamuni Buddha in SFD (Ming dynasty)	quartz, muscovite, albite, calcite
Tang-3	coarse clay layer	Bodhisattva Manjusri in XSBD (Tang dynasty)	quartz, muscovite, albite, calcite, chlorite
Tang-4		Bodhisattva Samantabhadra in XSBD (Tang dynasty)	quartz, muscovite, albite, calcite
Song-6		Sakyamuni Buddha in XSND (Song dynasty)	quartz, muscovite, albite, calcite, chlorite
Song-8		Bodhisattva Manjusri in XSND (Song dynasty)	quartz, muscovite, albite, calcite, chlorite
Ming-10		Sakyamuni Buddha in SFD (Ming dynasty)	quartz, muscovite, albite, calcite, chlorite

**Table 2:** Morphological characteristics of fibre samples in clay layer.

Sample No.	Position	Sculpture	Morphological Characteristics of Fibres	Fibre Identification
XSBD-F1	fine clay layer	Bodhisattva Samantabhadra in XSBD (Tang dynasty)	The single fibre is long (compared to wheat or rice straws) and shows uneven thickness along its length direction. There are obvious transverse knots, cell cavities and longitudinal stripes on the fibre wall.	Ramie
XSBD-F2		Bodhisattva Manjusri in XSBD (Tang dynasty)	The fibres are long (compared to wheat or rice straw) and appears in a dark wine red after staining. There are obvious transverse nodal lines on the fibre wall, and the diameter of the cell cavity is small, while the cavity itself is uneven. Both ends of the fibres are pointed, and the cell wall of the parenchyma cell is very thin, showing deformation and bending. Some translucent membranous tissue was found to connect with the parenchyma cells.	Kenaf
XSND-F4		Sakyamuni Buddha in XSND (Song dynasty)	The fibre is cylindrical and shows as a dark wine red after dyeing. The fibre shows uneven thickness along the length direction and has relatively thick cell walls. Fine transverse nodal lines are found on the cell wall.	Sisal hemp
XSND-F5		Bodhisattva Manjusri in XSND (Song dynasty)	The fibres are long and thin, showing a twisted shape. Its fibre wall is smooth without any knots or pits. Obvious cell cavities are visible, and the fibre appears wine red after dyeing.	Cotton
XSBD-F3	coarse clay layer	Bodhisattva Manjusri in XSBD (Tang dynasty)	The fibre is short, with sparse transverse knots. The cell cavity is obvious. Both ends of the fibre are blunt, with divergent or spherical ends. The fibre shows as blue purple after dyeing.	Green sandalwood bark

**Table 3:** Analysis of major colour pigments in the sculptures: VS (very strong), S (strong) and W (weak) represent the relative intensity of the Raman bands.

Sample No.	Name of Buddha	Colour	Main Elements (SEM-EDS)	Raman Bands (cm^−1^)	Pigment
SFD-33	Sakyamuni Buddha	Gold	Au, Ag, Pb, Cu, Fe, C, O	/	Gold (Au)
SJD-26	Ananda		Au, Ag, Pb, Cu, C, O	/	Gold (Au)
XSND-16	Bodhisattva Manjusri	Red	Pb, O, Si, Al, C, Ca, Fe	122(VS), 152(W), 227(W), 313(S), 392(S), 549(S)	Minium (Pb_3_O_4_)
SJD-29	Ananda		Pb, O, Si, Al, C, Ca	122(VS), 152(S), 231(W), 316(W), 392(S), 551(S)	Minium (Pb_3_O_4_)
XSND-17	Bodhisattva Manjusri		S, Hg, Pb, O, Ca, C, Si, Al, K, Fe	122(VS), 152(S), 225(W), 253(S), 314(S), 344(W), 392(S), 549(S)	Minium(Pb_3_O_4_) + Cinnabar(HgS)
XSND-13	Bodhisattva Manjusri		S, Hg, Pb, O, Ca, C, Cl, Si, Al, K, Fe	122(VS), 152(S), 226(W), 254(W), 314(W), 392(S), 549(S)	Minium(Pb_3_O_4_) + Cinnabar(HgS)
XSND-14	Sakyamuni Buddha		Fe, C, O, Si, Al, Ca, K	145(VS), 228(S), 294(S), 411(S), 1316(W), 1596(W)	Hematite (Fe_2_O_3_)
SJD-27	Sakyamuni Buddha		Fe, C, O, Si, Al, Ca	226(S), 293(VS), 412(S), 611(W)	Hematite (Fe_2_O_3_)
SFD-32	Sakyamuni Buddha		Fe, C, O, Si, Al, Ca, Cl	226(W), 294(S), 413(W), 1339(W), 1559(W)	Hematite (Fe_2_O_3_)
XSND-17	Bodhisattva Manjusri	Green	Cu, Cl, C, O, Si, Al, Ca, K	341(W), 418(S), 510(S), 834(W), 925(W), 3347(VS), 3434(VS)	Atacamite (Cu_2_(OH)_3_Cl)
SJD-20	Sakyamuni Buddha		Cu, Cl, C, O, Si, Al, Ca, Fe	219(S), 511(S), 824(W), 913(S), 977(S), 3345(VS), 3436(VS)	Atacamite (Cu_2_(OH)_3_Cl)
XSBD-3	Bodhisattva Manjusri		Cu, C, O, Si, Al, Ca	166(S), 349(S) 435(S), 577(W), 651(W), 751(W), 1078(S), 1355(S), 1492(S), 3374(S)	Malachite (Cu_2_(OH)_2_CO_3_)
SJD-24	Sakyamuni Buddha	Blue	Cu, C, O, Si, Al, Ca	253(S), 397(VS), 774(S), 839(S), 1355(S), 1589(S), 3430(S)	Azurite (Cu_3_(CO_3_)_2_(OH)_2_)
XSBD-4	Ananda	Yellow	Fe, C, O, Ca, S, Si, Al, K	144(VS), 247(S), 303(VS), 342(S), 393(VS)	Yellow ochre (FeO (OH)·nH_2_O)
SJD-22	Ananda	Black	C, O, Si, Al, Ca	1329(S), 1599(S)	Amorphous carbon (C)
XSBD-5	Maitreya buddha		C, O, Si, Al, Ca	1307(S), 1576(S)	Amorphous carbon (C)
XSBD-1	Bodhisattva Samantabhadr	White	Ti, C, O, Si, Al	146(VS), 400(S), 518(S), 641(S)	Titanium white (TiO_2_)
XSBD-2	Bodhisattva Manjusri		Si, Al, Ca, K, C, O	142(S), 404(S), 462(W), 1009(S)	Kaolinite+Gypsum (Ca(SO_4_)·(H_2_O)_2_)
XSBD-6	Bodhisattva Manjusri		Si, Al, Ca, K, C, O	143(S), 300(W), 403(W), 466(W), 1007(W)	Kaolinite+Gypsum (Ca(SO_4_)·(H_2_O)_2_)
